# Effects of the Newly Isolated T4-like Phage on Transmission of Plasmid-Borne Antibiotic Resistance Genes via Generalized Transduction

**DOI:** 10.3390/v13102070

**Published:** 2021-10-14

**Authors:** Junxuan Zhang, Xiaolu He, Shuqing Shen, Mengya Shi, Qin Zhou, Junlin Liu, Mianzhi Wang, Yongxue Sun

**Affiliations:** 1 College of Veterinary Medicine, South China Agricultural University, Guangzhou 510642, China; zhangjx20615@gmail.com (J.Z.); cherihe1124@gmail.com (X.H.); ssq0923224@163.com (S.S.); shimengya97@163.com (M.S.); eugenezhang403@gmail.com (Q.Z.); liujunlin448@gmail.com (J.L.); 2 National Risk Assessment Laboratory for Antimicrobial Resistance of Animal Original Bacteria, South China Agricultural University, Guangzhou 510642, China; 3 National Laboratory of Safety Evaluation (Environmental Assessment) of Veterinary Drugs, South China Agricultural University, Guangzhou 510642, China; 4 Guangdong Provincial Key Laboratory of Veterinary Pharmaceutics Development and Safety Evaluation, South China Agricultural University, Guangzhou 510642, China; 5 Guangdong Laboratory for Lingnan Modern Agriculture, Guangzhou 510000, China; 6 College of Veterinary Medicine, Yangzhou University, Yangzhou 225009, China; 7 Jiangsu Co-Innovation Center for Prevention and Control of Important Animal Infectious Diseases and Zoonoses, Yangzhou 225009, China

**Keywords:** lytic phage, antibiotic resistance genes, transducing particle, generalized transduction

## Abstract

Bacteriophages are the most abundant biological entities on earth and may play an important role in the transmission of antibiotic resistance genes (ARG) from host bacteria. Although the specialized transduction mediated by the temperate phage targeting a specific insertion site is widely explored, the carrying characteristics of “transducing particles” for different ARG subtypes in the process of generalized transduction remains largely unclear. Here, we isolated a new T4-like lytic phage targeting transconjugant *Escherichia coli* C600 that contained plasmid pHNAH67 (KX246266) and encoded 11 different ARG subtypes. We found that phage AH67C600_Q9 can misload plasmid-borne ARGs and package host DNA randomly. Moreover, for any specific ARG subtype, the carrying frequency was negatively correlated with the multiplicity of infection (MOI). Further, whole genome sequencing (WGS) identified that only 0.338% (4/1183) of the contigs of an entire purified phage population contained ARG sequences; these were *floR*, *sul2*, *aph(4)-Ia*, and *fosA*. The low coverage indicated that long-read sequencing methods are needed to explore the mechanism of ARG transmission during generalized transduction.

## 1. Introduction

Bacterial viruses or bacteriophages (phages) are the most abundant biological entities on earth and can be divided into lytic or temperate phages, according to their lifestyles [1,2]. Temperate phages integrate their DNA into the host genome and their lysogenic cycles enable vertical transmissions with the host DNA [3]. In contrast, infection by the lytic phage results in the direct cell lysis of the host and release progeny phage.

Packaging of phage genomes into intact virions also results in the incorporation of host DNA and generalized transduction is the process where host DNA is randomly assembled into progeny lysis and replaces either all or part of the parental phage genome [4]. This DNA can thereby be injected into recipient bacteria in the next round of infection and homologous recombination results in horizontal gene transfer (HGT) [5,6]. This process primarily occurs in the lytic phage [7,8]. Specialized transduction occurs in the temperate phage where phage integration occurs at specific genomic sites, such as prophage, which allows vertical transmission [9]. Activation of lysogenized prophage occurs under specific physical or chemical stressors, such as ultraviolet light or via intrastrand crosslinking with drugs, such as mitomycin C, and will induce the prophage to enter a lytic cycle. This process can result in incorporation of host DNA due to inaccurate cutting so that HGT events with antibiotic resistance genes (ARG) can occur in the next infection cycle by homologous recombination.

Conjugative transposons (CT) account for the majority of antibiotic resistance transfer within *Bacteroides* and contain genes reminiscent of transposons, plasmids, and bacteriophages and, thus, share characteristics with these other mobile DNA elements [10,11]. These processes of incorporation of host DNA are also mechanisms for ARG transfer and specialized transduction was shown to be a highly efficient mechanism for ARG transmission. For instance, the *mecA* gene of methicillin-resistant *Staphylococcus aureus* (MRSA) is located within a complex mobile genetic element, the SCCmec, which is a phage target since it can incorporate into chromosomal *att* sites [12,13]. Additionally, *Clostridium difficile* bacteriophage *ΦC2* transduces *erm*(B) via a novel transposon Tn6215 at frequencies as high as 10^−6^ [14]. The transduction frequency of genetic markers contained in *S. aureus* pathogenicity islands by phage 80α is 10^7^ times higher than when integrated at other sites [15]. Similarly, ARG distribution by porcine-derived enterotoxigenic *Escherichia coli* (ETEC) prophage indicated that host genomic sequences both upstream and downstream of ARGs contain highly conserved transposon and integron sequences, and most likely provide a powerful driving force for HGT events [16]. These cases illustrate the significant role for specialized transduction in ARG spread.

Interestingly, both lytic and temperate phages incorporate ARGs at frequencies greater than expected if the events are absolutely random [6,17]. The types of sequences that can be transferred by specialized transduction seem to be restricted; for this reason, it appears that specialized transduction is a minor contributor to gene transfer in the environment compared with generalized transduction [5]. Phage particles carrying ARGs have been reported in different biomes, including sludge [18], soil [19], wastewater [20,21], marine viromes [22], and human and animal biomes [23,24]. The high persistence of phages in the environment enhance the efficiency of ARG dissemination in phage particles, which could, in turn, increase the chances of ARG transduction [22]. The offspring phages are capable of injecting the bacterial genes into next recipient cell, which can subsequently be incorporated into the host genome by recombination [21,25]. The use of ARGs by phage may not entirely be the result of errors in DNA packaging; if selected by phages, these genes can improve their inclusive fitness and ensure their survival [26].

In this study, we isolated the T4-like lytic phage from pig farm sewage that targeted an *E. coli* C600 transconjugant containing the plasmid pHNAH67. We characterized 11 ARG subtypes to assess the carrying frequencies of transducing particles at different multiplicity of infection (MOI) groups to explore whether generalized transduction plays a role in ARG transmission. We also utilized whole genome sequencing (WGS) to examine the genomic environments (or accessory genome) of the packaged ARGs in an attempt to reveal mechanisms of the dynamic nature in the packaging of these ARGs.

## 2. Materials and Methods

### 2.1. Strains and Plasmids

**Figure 1:**
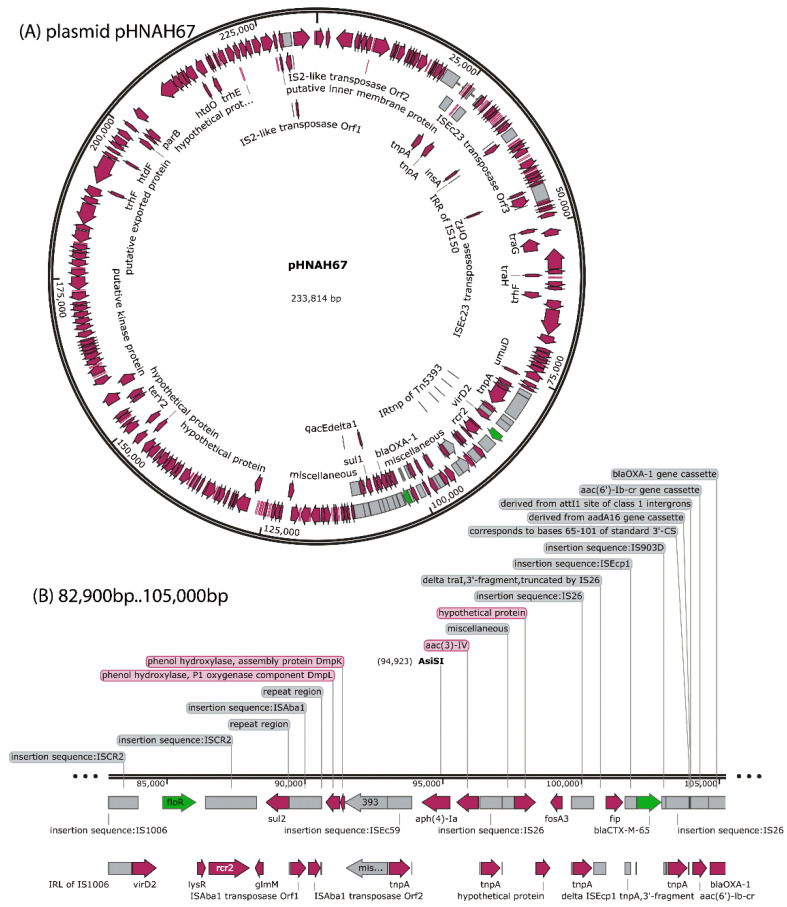
Gene map of lncHI2 plasmid pHNAH67. (**A**) Full annotated plasmid map. (**B**) Partial map bp 82,900 to 105,000 indicating the positions of ARG subtypes *floR*, *sul2*, *aph(4)-Ia*, *acc(3)-IV*, *fosA3*, *bla*_CTX-M-65_, *aac(6′)-lb-cr*, *bla*_OXA-1_, *catB3*, *arr3,* and *sul1*.

The lncHI2 plasmid pHNAH67 (NCBI sequence number: KX246266) possessed the following ARGs: *floR*, *sul2*, *aph(4)-Ia*, *aac-(3)-IV*, *fosA3*, *bla*_CTX-M-65_, *aac(6’)-lb-cr*, *bla*_OXA-47_, *catB3*, *arr3,* and *sul1* (Figure 1). The lncHI2 plasmid pHNAH67 was transferred into *E. coli* C600 to obtain the transconjugant AH67C600, a single colony isolated from a streak culture of an Luria–Bertani (LB) plate. Bacteria were cultivated in LB liquid medium at 37 °C with shaking at 160 rpm until early log phase growth.

### 2.2. Acquisition of Phage

The lytic phage AH67C600_Q9 was isolated from a wastewater sample obtained from a pig farm sewage treatment system through additional pretreatment steps [27,28]. In brief, the sewage samples were concentrated by an ultrafiltration device (Millipore Labscale tangential flow filtration System, Merck KGaA, Darmstadt, Germany) and then filtered through a 0.22 µm membrane (Millex-GP, Millipore, Burlington, MA, USA) [29]. The filtrate was incubated with logarithmic phase cells of *E. coli* AH67C600 overnight at 37 °C with shaking at 160 rpm. The mixture was centrifuged and filtered as per above and used to inoculate new bacterial cultures to enrich the primary phage preparation. The final filtrates were plaque-purified using the double-layer soft agar plate method [30,31]. Single plaques were selected and diluted in SM buffer (100 mM NaCl, 8 mM MgSO_4_, 50 mM Tris-HCl pH 7.5) and stored at 4 °C. The single phage was plaque-purified three times by *E. coli* C600 before use and standardized titers in the range of 10^7^–10^9^ plaque forming units (PFU) per mL were used for follow-up experiments.

### 2.3. Identification of Phage

#### 2.3.1. Transmission Electron Microscopy

Electron micrographs of purified phage particles were obtained as previously described [32,33]. High titer phage stocks were concentrated 50-fold using 100 kDa Amicon Ultra centrifugal filter units (Millipore, Burlington, MA, USA) and 15 μL of phage concentrate was dropped on carbon-coated formvar covered grids and left to stand for 15 min. The preparations were then stained with 2% *w/v* phosphotungstic acid pH 7.0 and air-dried. The phages were examined using an FEI transmission electron microscope (Thermo Fisher, Hillsboro, OR, USA) at an acceleration voltage of 80 kV.

#### 2.3.2. Optimal Multiplicity of Infection

Bacterial colony forming units (CFU) per mL and phage titers (PFU per mL) were determined as previously described [34]. Optimal multiplicity of infection (OMOI) was examined by using appropriate susceptible host cultured to early log phase growth and incubated with the phage for 2 h in the following ratios: phage AH67C600_Q9: host bacteria AH67C600 = 10:1, 1:1, 0.1:1, 0.01:1, 0.001:1 and 0.0001:1. Then phage titers were calculated again in each group using standard protocols in triplicate [27].

#### 2.3.3. One Step Growth Curve

One-step growth experiments were carried out as described previously with a few modifications [35,36,37]. In brief, the phage AH67C600_Q9 was added at the MOI of 10 and allowed to absorb to *E. coli* AH67C600 cells for 10 min at room temperature. The mixture was centrifuged at 2700× *g* for 10 min to remove unabsorbed phages and infected cells were resuspended with LB to 5 mL. The culture samples were harvested every 10 min and phage numbers were quantified as per above. This experiment was performed in triplicate.

#### 2.3.4. Physical Stability of the Phage

Phage thermostability was examined by incubating high titer phage stocks (≥10^8^ PFU per mL) at 4, 37, 50, 55, 60, and 70 °C for 60 min and pH tolerance was examined by incubating over a pH range of 1.0 to 12.0 for 60 min at 37 °C, as previously described, with a few modifications [38,39]. Phage titers were determined by the double-layer agar plate as per above. Both experiments were performed three times.

### 2.4. Bacterial and Bacteriophage DNA Extraction

Bacterial and plasmid DNA were extracted using Bacterial DNA Kit and Plasmid Mini Kit (Omega Bio-Tek, Norcross, GA, USA), according to the recommendations by the manufacturer. The purified phage lysates were concentrated by polyethylene glycol (PEG8000) and treated with DNase I (Sangon Biotech, Shanghai, China) at 100 U per mL prior to DNA extraction. Phage DNA extraction was performed according to published methods [27,28,40]. An aliquot of a phage lysate was amplified for bacterial 16S rDNA determinations using conventional polymerase chain reaction (PCR) and only negative samples were used for the subsequent sequencing [17].

DNA was suspended in TE buffer (10 mM Tris-HCl, 1 mM EDTA, pH 7.5) and DNA concentrations were determined by UV spectroscopy with an ND-1000 instrument (NanoDrop, Wilmington, NC, USA).

### 2.5. Lytic Phage Genome Sequencing and Assembly

Because the classification of the isolated phage was unknown, we performed de novo genome sequencing. In brief, phage DNA samples were sent for de novo sequencing to GENEWIZ Biological Technology (Suzhou, China) and a library of these DNA samples that had passed the inspection was constructed for cluster preparation and sequencing. Bcl2fastq (v.2.17.1.14) was used to perform image-based recognition (Base Calling) on the original image data. After quality analysis, the raw sequencing data were obtained. Trimmomatic-0.36 was used to trim and remove adaptors and low-quality nucleotides from the original readings to obtain clean data for subsequent information analysis [41]. Spades Genome Assembler 3.8.1-Linux version was used to assemble the post-processed readings into contig sequences [42]. SSPACE (v.3.0) was used to further assemble contig sequences into scaffold sequences and GapFiller (v.1.10) was used to complement and extend the scaffold sequence to obtain the final scaffold sequence of the whole phage genome.

Three online websites, Open Reading Frame Finder (www.ncbi.nlm.nih.gov/orffinder/, accessed on 29 February 2020, RASTtk (https://rast.nmpdr.org/, accessed on 29 February 2020) and GeneMarks (http://topaz.gatech.edu/GeneMark/, accessed on 29 February 2020), were jointly used to predict all genes in the genome [43,44,45]. After comparisons with protein sequences of known function for similarity and identity, the best hit was selected for the annotation of the gene products. The manual search method was also used to locate ARGs and virulence genes in the genome using the online websites VFDB and ARDB. Finally, CGview (v.1.0) was used to draw the whole genome map of the phage.

All uploaded phage genome sequences in NCBI were downloaded to compare with the isolated phage genome sequences and amino acid sequences by local BLAST+ (v.2.7.1). The genome alignment map of the isolated phage was drawn in Easy-fig; the gene encoding products with different functions are indicated with different colors.

The evolutionary relationships between phages were analyzed using phylogenetic trees based on the amino acid sequences of the terminase large subunits of different Escherichia phages. The phage terminase is a unique protein of the dsDNA phage and possesses highly conserved functions to drive packaging by energizing the DNA [46]. Terminase of the double-stranded DNA phage can be divided into a large subunit and a small subunit according to their molecular weight [47]. The large subunit has endonucleolytic and ATPase activities and is generally composed of 400 to 750 amino acids [48]. After aligning the sequences of the terminase large subunit, similar sequences were downloaded from the National Center for Biotechnology (NCBI). Phylogenetic analysis was performed with MEGA7 by the neighbor-joining method (Bootstrap: 1000 with the Poisson Model) [49]. Alignment analysis was based on the ClustalW alignment of amino acid sequences by MEGA7 (parameter setting: Gap Opening Penalty: 10, Gap Extension Penalty: 0.2) [50].

### 2.6. Effect of Lytic Phage Generalized Transduction on Plasmid-Borne ARGs

We chose 11 ARGs (carried on plasmid pHNAH67 as the research object) to evaluate phage misloading values, which represented the carrying frequency of transducing particle in generalized transduction. The primers for these ARGs in real-time quantitative polymerase chain reaction (qPCR) analysis are shown in Table S1. Published articles have described in detail how to establish a standard curve to quantify the copy numbers of the investigated genes [40,51,52]. In brief, positive amplification products were purified with a Gel Extraction Kit (Omega Bio-Tek, Norcross, GA, USA) and ligated into the pMD19-T Vector (Takara, Dalian, China). Recombinant plasmid vectors were amplified in bacterial host *Escherichia coli* DH5α (Takara). The extracted plasmid samples were sequenced (Tsingke Biotechnology, Beijing, China) and analyzed using the BLAST website at NCBI (https://blast.ncbi.nlm.nih.gov/Blast.cgi, accessed on 23 August 2020) (Table S2). Ten-fold serial dilutions of plasmid-cloned genes were used to construct qPCR standard curves for the 11 ARGs (Table S3).

Three experimental groups were set (MOI = 10:1, 0.1:1, 0.001:1) to determine whether the ratio of phage and the host affects the misloading value for the different ARGs. The carrying frequency was calculated using Equation (1), which determines the frequency for the packaging of an ARG, expressed as the ratio of the absolute abundance of the ARG in the progeny phage and the numbers of the parent phage.

(1) $$\mathrm{Misloading}\;\mathrm{value}=\frac{\mathrm{Absolute}\;\mathrm{Abundance}\;\mathrm{of}\;\mathrm{ARGs}\;\mathrm{in}\;\mathrm{Progeny}\;\mathrm{Phage}\;\mathrm{Genome}\;(\mathrm{Copies})}{\mathrm{Number}\;\mathrm{of}\;\mathrm{Parent}\;\mathrm{Phage}\;(\mathrm{PFU})}$$

In the process of generalized transduction, the “misloaded phage” mixed in the offspring retain infectivity only for the recipient host bacteria without the ability to regenerate and re-lyse; this makes their isolation difficult using traditional methods. Therefore, we incubated the phage AH67C600_Q9 and host bacteria AH67C600 at the MOI of 1, and extracted the phage genome as described above. Then, Illumina hiseq4000 (Genewiz, Suzhou, China) was applied for deep sequencing (PE150 Mate-Pair Sequencing) and Spades Genome Assembler 3.8.1—Linux version was used for offline data to assemble post-processed readings into contigs [42]. Local BLAST+ was used to compare contigs with the 11 ARGs carried on the plasmid pHNAH67; the upstream and downstream sequences were extracted to analyze the potential impact on the propagation of ARGs in lytic phage transduction. Bowtie2 (v.2.4.4), SAMtools (v.1.7), and deepTools (v.3.5.1) were used to convert sequencing data into bigwig format. Integrative Genomics Viewer (IGV, v.2.11.1) was used to construct mapping analysis using log scale [53].

## 3. Results

### 3.1. Isolation and Characterization of Lytic Phage

**Figure 2:**
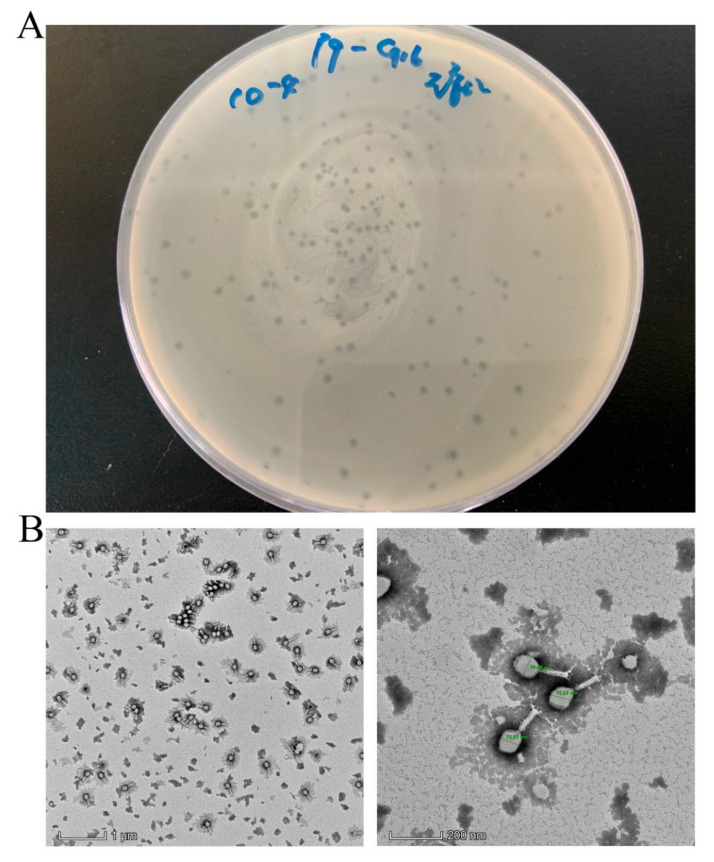
Morphological characteristics of phage AH67C600_Q9. (**A**) Culture map of phage AH67C600_Q9 plaques in double-layer agar. (**B**) TEM images of phage AH67C600_Q9. The longitudinal diameter of the capsid approximately equaled the tail length.

A lytic phage was isolated from a pig farm sewage treatment pond in Huizhou, China, named AH67C600_Q9, which was able to lyse *E. coli* AH67C600. Phage AH67C600_Q9 formed transparent, small, and round plaques of approximately 1.0 mm in diameter on a lawn of *E. coli* AH67C600 (Figure 2A). TEM observations of the phage revealed an icosahedral capsid with a cross diameter of 76.94 ± 2.40 nm (*n* > 10), and the longitudinal diameter of capsid was 100.39 ± 4.26 nm (*n* > 10). The tail length was 103.05 ± 3.30 nm (*n* > 10) (Figure 2B). This phage could therefore be classified morphologically in the order Myoviridae and the family Caudovirales.

### 3.2. Optimal Multiplicity of Infection of Lytic Phage

**Figure 3:**
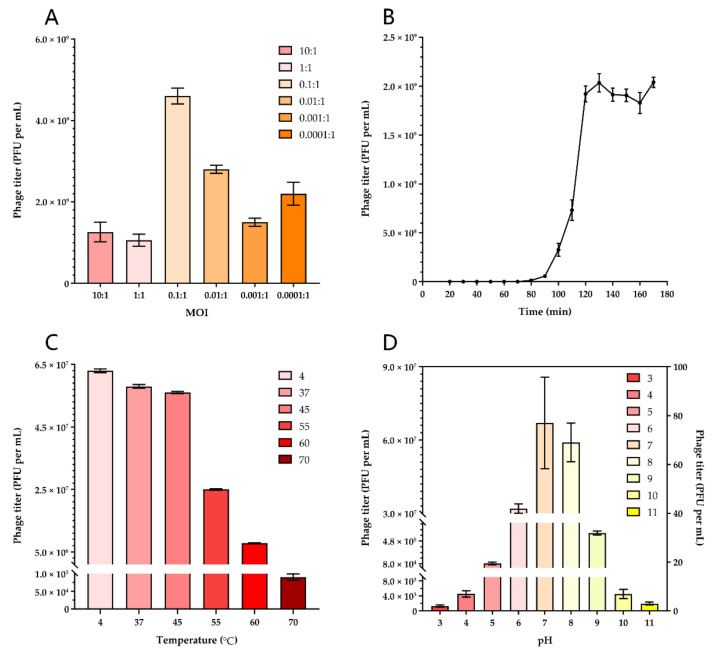
Physiological characteristics of phage AH67C600_Q9. (**A**) Optimal MOI test. The phage AH67C600_Q9 achieved the highest titer after cultured while the MOI is 0.1:1 (4.63 × 10^8^ PFU per mL). (**B**) One step curve of phage AH67C600_Q9. After 20 min of early treatment time, 20 to 80 min is the latent time and 80 to 120 min is the burst time. At the plateau phase, the maximum number of progeny was 90 PFU per cell. (**C**) Thermal stability test. The phage AH67C600_Q9 had stable biological activity at temperatures of 4 to 50 °C. (**D**) pH stability test. The phage AH67C600_Q9 is stable at the pH of 6 to 8. Due to the large difference between results, titers for pH = 3, 4, and 11 are listed on the right *y*-axis and the rest are on the left *y*-axis.

We tested the MOI ranging from 10 to 0.0001 to determine the optimal values for producing maximal phage titers. When the ratio of the phage AH67C600_Q9 and host AH67C600 was 0.1, the titer was maximal at 4.63 × 10^8^ PFU per mL and this optimal MOI was used to guide follow-up experiments (Figure 3A).

### 3.3. One-Step Growth Curve

The one-step growth curve for the phage AH67C600_Q9 was analyzed by infecting the bacterial host during the exponential growth phase at an MOI of 10 to ensure complete culture lysis without bacterial regrowth. The phage AH67C600_Q9 had a latent period of 50 to 60 min and a burst period of 40 min. These conditions produced the maximum number of progeny at 1.80 × 10^9^ PFU per mL with an average of 90 PFU per cell (Figure 3B).

### 3.4. Phage Stability Tests

The thermostability and pH tolerance of the phages were analyzed; incubations at 4 and 37 °C for 60 min did not alter the titers. In contrast, the titer gradually decreased with incubations at 45, 55, and 60 °C; at 70 °C the titers decreased about 3 logs (Figure 3C). The titer was also stable from pH 6 to 8 after 60 min incubation. At pH 5 or 9 for 60 min, the titers decreased by 2–3 logs, and at pH 10, by 4 logs. The phage had little activity in the pH ranges of 1 to 4 and 11 to 12 (Figure 3D).

### 3.5. Sequencing and Bioinformatics Analysis of Phage AH67C600_Q9 Genome

**Figure 4:**
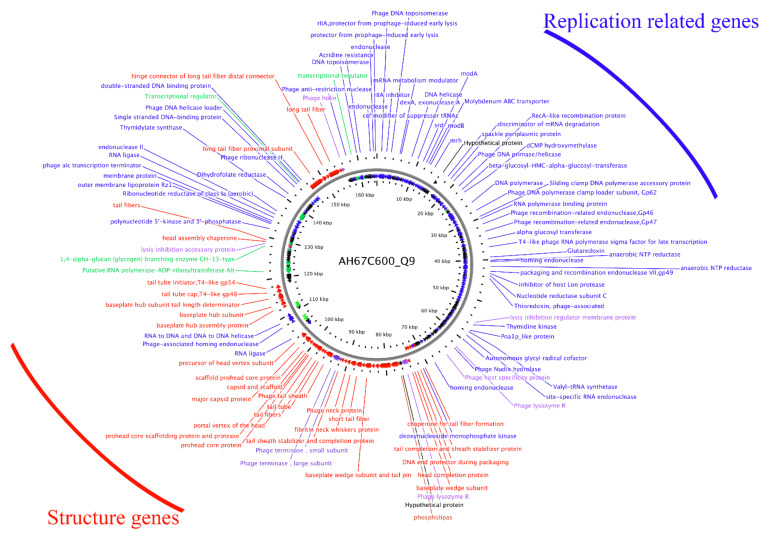
Annotation of the predicted genes of phage AH67C600_Q9. The genes located at upper right are replication-related and the structural genes are concentrated in the lower left corner. Blue, replication related genes; red, structure genes; purple, lytic related; green, transcription related; black, hypothetical genes.

**Figure 5:**
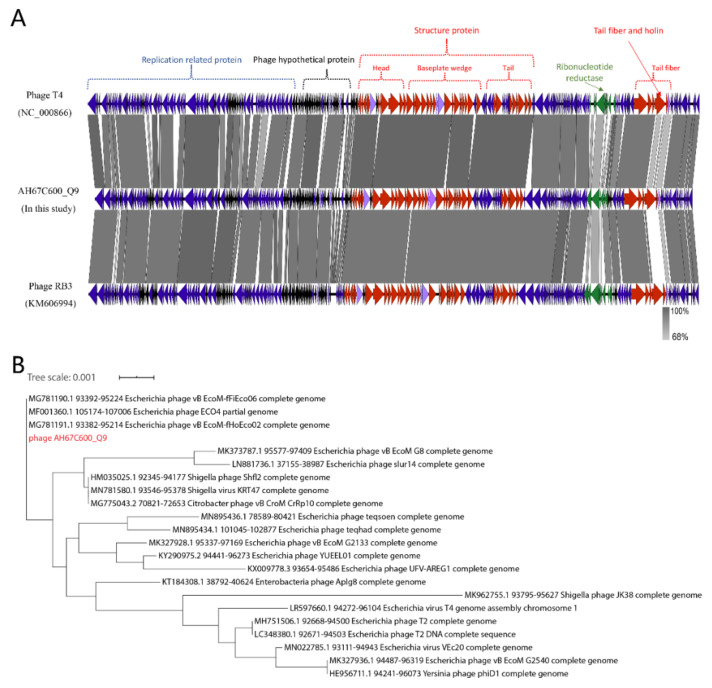
(**A**) Genome sequence alignment of phage AH67C600_Q9, T4 and RB3. The functional modules are indicated by color and similarities are shown in gray, according to the scale on the bottom right corner. (**B**) Phylogenetic analysis based on the large subunit of terminase for *Enterobacteriaceae* phage, demonstrating an independent branch for phage AH67C600_Q9.

We completely sequenced the genome of phage AH67C600_Q9 and found it was 164,663 bp with a GC content of 35.62% with 264 predicted genes (Figure 4). The phage was most similar to the Enterobacteria phage RB3 (acc. no. KM606994) with 89% query coverage in local BLAST+. In addition, since the morphology of the phage AH67C600_Q9 and Enterobacteria phage T4 (acc. no. NC_000866) were structurally similar, we compared the three phage genomes (Figure 5A). The sequence for AH67C600_Q9 was submitted to GenBank under accession number MZ681930.

The phage AH67C600_Q9 genome showed a typical modular arrangement in its structure. The distribution of genes possessing similar functions from other similar phages were located at almost identical sites in the genome with slight differences between gene products. The structural protein sequence of phage AH67C600_Q9 isolated in this study was highly similar to the phage T4 and RB3, indicating extremely conservative structural characteristics, and was consistent with the TEM results (see above). The replication and transcription proteins were also highly similar to the other phages and only minor differences in the gene products were observed. According to Figure 5A, we compared the amino acid sequences of tail fiber and ribonucleotide reductase with lower homology (Table S4). In amino acid sequence alignment, four of the eight predicted tail fiber genes possessed identities <95% to the Enterobacteria phage RB3 (Figure S1). The differences in the amino acid sequence of the tail fibers between the three phage most likely reflected the differences in the respective host ranges [54]. Additionally, three of four ribonucleotide reductase gene products were <95% identical to Enterobacteria phage T4, indicating a novel T4-like phage [55]. As a lytic protein, the amino acid sequences of holin were only about 75% identical between three bacteriophage. Similarly, the phage AH67C600_Q9 was located in an independent branch in the phylogenetic tree that was constructed based on the amino acid sequences of the terminase large subunits. Although AH67C600_Q9 was closely related to the T4-like phage, it differed greatly from the prototype phage T4 in evolution and had a different evolutionary relationship with phage RB3 (Figure 5B).

### 3.6. Lytic Phage Misloaded Plasmid-Borne ARGs by Generalized Transduction

**Figure 6:**
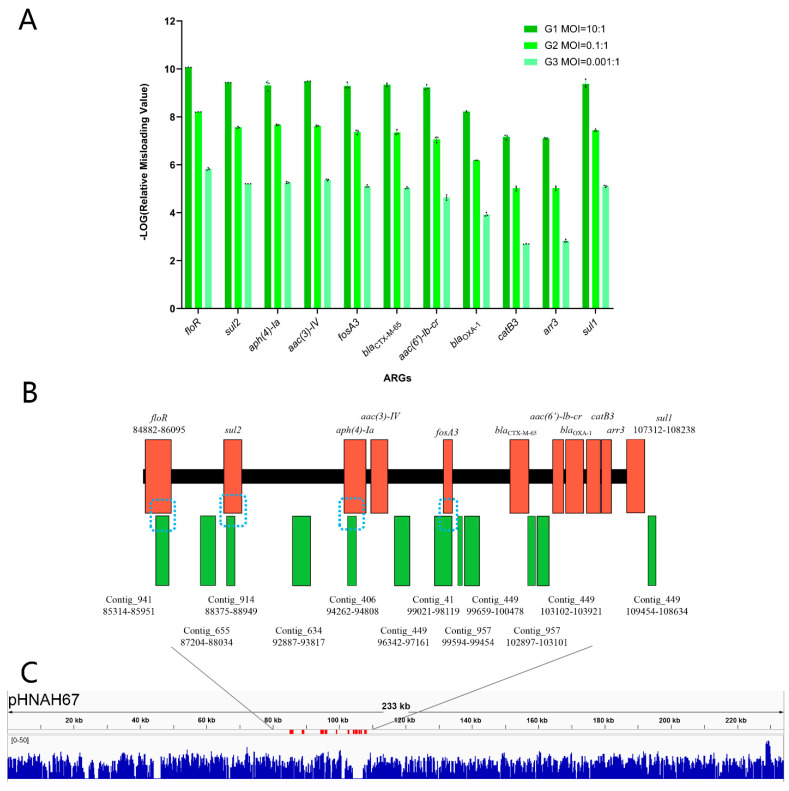
(**A**) Relative misloading value of 11 ARGs in “misloaded phage”. Results for qPCR assays indicated differences for ARG transmission through generalized transduction in three groups. It is worth noting that the *y*-axis represented the negative logarithm of relative misloading value. (**B**) Matching positions of ARGs between contigs and plasmid pHNAH67. The blue dotted frames indicate the alignment of four contigs and ARG sequences. (**C**) Plasmid pHNAH67 genome coverage pattern associated with generalized transducing by phage AH67C600_Q9. Whole genome sequencing reads were mapped to plasmid in blue. The red regions on the sequence are the 11 ARGs.

In the natural environment, phage–host interactions are not carried out under the optimal MOI. We determined whether the MOI affects the frequency of ARG incorporation into the phage and termed this the misloading value. The absolute abundance of the 11 ARGs carried on the plasmid pHNAH67 differed in qPCR assay (Table S3), so we divided the misloading value of each ARG by the absolute abundance of this gene in the plasmid pHNAH67. This was defined as the relative misloading value. In order to facilitate the graphic representations, the relative misloading value was negative log transformed (Figure 6A). We found that the misloading value and MOI were negatively correlated. Contrary to the initial phage titers in the three experimental groups, the relative misloading values of each specific ARG subtype showed a 100-fold increase.

In generalized transduction, phage particles containing host DNA most likely would be incapable of producing another round of a productive infection. We analyzed phage content following infection of the host bacterium containing the 11 ARGs using WGS and obtained 6,927,578 raw data reads and 1183 contigs (Table S5). The reads for the genes *floR*, *sul2*, *aph(4)-Ia*, and *fosA* were all >20, even though the highest proportion of total reads was only 4.6 per 100,000 (*floR*). An analysis of the assembled contigs found that a total of 12 contigs matched the ARG sequences, but only 4 contigs contained ARG sequences. These were contig_941_655bp (*floR*), contig_914_575bp (*sul2*), contig_406_625bp (*aph(4)-Ia*), and contig_41_903bp (*fosA*) (Figure 6B). However, these contigs only contained ARG fragments and complete ARG sequences could not be successfully located from the progeny phage using next-generation sequencing [56]. Additionally, the reads were distributed on the plasmid sequence randomly, indicating the randomness of misloading events in lytic cycle (Figure 6C). The ARG with higher absolute abundance on the plasmid pHNAH67 has more opportunities to be misloaded.

## 4. Discussion

Phages are the most abundant and diverse organisms on earth and exert major influences on the diversity of bacterial communities [57]. In particular, phages can transmit function-related genes through transduction to help the hosts adapt to their surroundings; these include ARGs and virulence genes [58]. However, although phages are widely distributed, there may be phages that cannot be recovered using standard laboratory approaches or are missed by this approach [59]. In this study, wastewater samples were collected from a sewage treatment pond of a large-scale pig farm in Huizhou, China. We isolated the lytic phage AH67C600_Q9 by plaque purification and it formed transparent plaques, indicating it was a lytic phage. The head of phage AH67C600_Q9 was a typical viral particle structure similar to the T4 phage in the family Myoviridae and order Caudovirales [60]. The optimal MOI of AH67C600_Q9 was determined to be 0.1:1. The latent time was 50 to 60 min with a burst time of 40 min and lysis was completed at 120 min at about 90 PFU per cell. The phage was stable at pH 6 to 8 at 4 to 37 °C showing nearly perfect survival ability in the natural environment.

The overuse of antibiotics has brought about major increases in bacterial resistance and has become a major global health threat. The role of phages in the transfer of antibiotic resistance has not been studied in depth. No ARGs or virulence genes were found in the genome of the lytic phage AH67C600_Q9 (Figure 4). The structural distribution of genes included lysis, replication, and regulatory proteins in the forward sequence and structural proteins on the opposite DNA strand. The distribution of genes in its annotation map were similar to known T4 phage and published T4-like phages, such as Enterobacteria phage RB3, indicating a close relationship with the T4 family. These phage genomes were highly similar, except for the tail fiber, ribonucleotide reductase, and holin (Figure 5A). The differences in the amino acid sequence of the tail fiber most likely reflect the diversity of host specific [54]. Ribonucleotide reductase possessed the unique ability to catalyze the reduction of all four ribonucleotides to the corresponding deoxyribonucleotides and is a rate-limiting enzyme for DNA synthesis and repair. The usual case is that amino acid sequence similarities for ribonucleotide reductase between organisms is remarkably low, although the active centers in the tertiary structure are conserved [61]. The lytic phage AH67C600_Q9 differed from the prototypical phage T4 in three ribonucleotide reductase amino acid sequences, indicating that the lytic phage AH67C600_Q9 may be a new type of T4-like phage with specific functions and environmental survival properties. The holin-lysozyme system has a broad spectrum of lytic activity against bacteria and independently can hydrolyze bacterial cell wall peptidoglycan, and as such plays a key role in cell lysis [62]. The holin sequence for AH67C600_Q9 differed from the prototypical T4 phage at 75.34 percent identity, suggesting that the holin-like protein may have different bactericidal performances and is worth further study [63].

Overall, the location of other functional genes in the genome of this new phage displayed only slight differences in a few predicted genes from the prototypical T4 phage; this was also reflected in its structure in TEM photomicrographs. However, phage AH67C600_Q9 was located on an independent branch of the phylogenetic tree established by the conserved sequence of the terminase large subunit in the Enterobacteriaceae phage. Although phage AH67C600_Q9 belongs to the T4-like phage, it differs greatly from the prototypical T4 in evolution as well as from RB3. This indicated that phage AH67C600_Q9 was a novel T4-like phage.

Bacteria and their viruses interact beneficially and antagonistically in polymicrobial communities, leading to ARG transmission during transduction that may hinder phage-based therapy [64]. Generalized transduction can result in the erroneous assembly of portions of the host genome into its progeny during the lysis cycle. These misloaded phages can therefore transfer this specific content to the recipient bacteria at the next infection by homologous recombination. In this process, the specific transposon structure in the upstream and downstream ARG sequences may be an additional mechanism ARG transmission. This also implies that the primary sequence of the ARGs or the genetic environment of the phage itself significantly affects the ARG transmission [16].

Transduction frequency is used to evaluate the probability of transmission of an ARG through phage transduction [14,65,66]. After co-cultivation of phage and host bacteria carrying specific ARGs, the resulting purified progeny phage are then used to assess the final probability of an ARG being transmitted from a specific donor bacterium to a specific recipient bacterium. However, some ARGs were transferred at a higher frequency than others. This indicated that the ease of homologous recombination within the recipient host bacterial was the primary determinant of the transduction frequency. Therefore, it does not represent all of the possibilities in nature and does not represent the possibility of the transduction of a certain gene. In the analysis of numerous samples, ARG transfer frequencies differed between ARGs, indicating a regular instead of a random process [17]. In our study, the relative misloading values were opposite to the decrease of the initial phage titer, maintaining an overall 100-fold increase, so the MOI could not be a fundamental factor in ARGs transmission by generalized transduction. In the same incubation time, with lower MOI, phage has more generations in the culture, which improves the opportunities of misloaded events. During the packaging process, the chances of different host genes being misloaded are overall the same. Misloaded phages can transfer genetic information in the next infection, which promote rapid evolution of prokaryotic genomes [4]. As previously documented, the genetic environment of a particular ARG could play an important role in this form of ARG transmission; transposons located upstream and downstream of an ARG most likely has a profound influence on the frequency of ARG transmission by generalized transduction [5]. As shown in Figure 6C, plasmid pHNAH67 was randomly packaged into the misloaded phage heads. T4-like phages use a *pac* sequence to initiate the DNA packaging series, and generalized transduction relies on the existence of *pac* site homologs scattered around the bacterial sequence [47,60,67]. The potential transfer of genes mediated by lytic phages should be considered important, in particular, the genes mobilized can affect human and animal health (including ARGs and virulence genes) [5]. As a widespread mechanism common to most bacteria, generalized transduction can transfer any gene from one bacterium to another [4].

The transfer of host DNA by generalized transduction lacks the genetic component necessary for cell lysis so the phage containing ARGs cannot be isolated by phage amplification procedures. Instead, we used WGS to analyze whole phage population and generated 6,927,578 raw reads. After assembly, 1183 contigs were included and 4 contained ARG sequences: contig_941_655bp (*floR*, 1213 bp), contig_914_575bp (*sul2*, 815 bp), contig_406_625bp (*aph(4)-Ia*, 1025 bp), and contig_41_903bp (*fosA*, 416 bp). Despite this, the ARG genetic environments were not represented in full since the contigs sequence was shorter than the full length of the gene, except for *fosA,* and the latter still failed to meet the demands of subsequent analysis. Therefore, there data were not sufficient to speculate on this form of ARG transmission by high-throughput sequencing or to use sequence assembly into contigs to retrospectively analyze the upstream and downstream genetic environment of ARGs [56]. For the inability to effectively search for ARGs and their upstream and downstream gene loops by deep sequencing and assembling into contigs, a possible explanation is that it is extremely difficult to locate the small numbers of an ARG-positive phage in the phage progeny. We found only 32 reads that matched the entire *floR* gene in a total of 6,927,578 reads, 16 of which were not 100% matched and were single reads of 150 bp. Perhaps increasing the original concentration of the phage and increasing the sequencing depth will be an effective solution, but existing experimental conditions cannot guarantee the removal of bacterial genetic material after lysis, and may increase the contamination of bacterial genetic material. Consequently, it is of great significance to study new methods to increase the separation capacity of these transducing phages that carry ARGs.

## 5. Conclusions

In this study, we isolated and characterized the lytic phage AH67C600_Q9 from a sewage treatment pond in a large big pig farm in Huizhou, China. The T4-like phage was a novel member of the family Myoviridae and the order Caudovirales and phage AH67C600_Q9 was located at an independent branch of the phylogenetic tree established by the conserved sequence of the terminase large subunit in the Enterobacteriaceae phage. Phage AH67C600_Q9 was able to misload host DNA, including plasmid-borne ARGs, and the carrying frequency was negatively correlated with MOI for any specific ARG subtype.

Although generalized transduction carried out by the lytic phage is an important part of horizontal gene exchange, our experimental conditions were not able to determine whether intact ARGs could be transferred by generalized transduction. The isolation and identification of lytic phage AH67C600_Q9 and the potential effects on ARG transmission in this study will lay a foundation for the follow-up study on transduction mechanisms of ARGs by phages.

## Supplementary Materials

The following are available online at https://www.mdpi.com/article/10.3390/v13102070/s1, Table S1: PCR primers used in this study, Table S2: BLAST results for pMD20-T:: ARG, Table S3: Conditions and results of qPCR for 11 ARGs, Table S4: Results of amino acid sequence alignments of tail fiber, ribonucleotide reductase and holin between Enterobacteria phage T4 or RB3 and phage AH67C600_Q9, Table S5: Matching positions of contigs to ARGs for *E. coli* AH67C600, Figure S1: Amino acid sequence alignments of tail fiber, ribonucleotide reductase and holin between Enterobacteria phage T4 or RB3 and phage AH67C600_Q9.
