# Supplementary material for: Effects of the Newly Isolated T4-like Phage on Transmission of Plasmid-Borne Antibiotic Resistance Genes via Generalized Transduction
Source: Viruses. 2021 Oct 14;13(10):2070. doi: 10.3390/v13102070 (PMC8538795; doi:10.3390/v13102070)
Supplement: Supplementary file 1 [file viruses-13-02070-s001.zip › viruses-1400745-supplementary.pdf]

viruses

MDPI

Supplementary Materials

Effects of the Newly Isolated T4-like Phage on Transmission of Plasmid-Borne Antibiotic Resistance Genes via Generalized Transduction

Table S1. PCR primers used in this study.

Gene	Primer Sequence (5′-3′)	AS[a]	MT[b]	Plasmid Position
floR	F-CGATGCTCGCACTCCTAA<br>R-GGCAAAGCTGAATCCGAT	224	55	85417-85640
sul2	F-CGGTTGCGTTTGATACCG<br>R-ACGCAAGCCTATGCCTTG	239	55	88906-89144
aph(4)-Ia	F-TTGTTGGAGCCGAAATCC<br>R-CCGATCTTAGCCAGACGA	235	55	94679-94913
aac(3)-IV	F-AATCGACGCGTACCAACT<br>R-CACAGGCAGAGCAGATCA	222	55	95745-95966
fosA3	F-CCGTCAGGGTCGAGAAAA<br>R-TGGGATAGCGGAGCCTAT	227	55	99011-99237
bla$_{CTX-M-65}$	F-AGACGTTGCGTCAGCTTA<br>R-GCGGCTGGGTAAAATAGG	237	55	102623-102859
aac(6′)-lb-cr	F-AATGCTGAATGGAGAGCC<br>R-GGTCCGTTTGGATCTTGG	218	54.4	104207-104424
bla$_{OXA-1}$	F-GCCAGTGCATCAACAGAT<br>R-CCATTCCTTTGGGGGTTT	241	55.6	104764-105004
catB3	F-AATCAGGGGCATCGGTAC<br>R-CCGCCAACGATAGCGTAA	239	57.8	105891-106129
arr3	F-CGAGGACGGTCGTATTCT<br>R-CAAGGGTTCGCAGGTTCT	214	55	106508-106721
sul1	F-CTGCGCTCTATCCCGATA<br>R-TGCGGGGCTCAAGAAAAA	224	55	107724-107947

The PCR procedure was as follows: initial 95 °C denaturation for 2 min followed by 35 cycles consisting of 95 °C for 10 s, annealing for 30 s at the melting temperatures shown above, and extension at 72 °C for 1 min and a final step at 72 °C for 10 min. [a]Amplicon size (bp). [b]Melting temperature (°C).

Table S2. BLAST results for pMD20-T:: ARG.

Gene	Length(bp)	NCBI Acc. No	Match rate (%)
floR	224	CP047011	100%
sul2	239	MW535748	100%
aph(4)-Ia	235	CP046417	100%
aac(3)-IV	222	CP046417	100%
fosA3	227	CP047005	100%
bla$_{CTX-M-65}$	237	MW052535	100%
aac(6′)-lb-cr	218	KJ568510	100%
bla$_{OXA-1}$	241	MW527089	100%
catB3	239	CP054408	100%
arr3	214	MW521224	100%
sul1	224	MZ275239	100%

Table S3. Conditions and results of qPCR for 11 ARGs.

Gene	qPCR conditions				Absolute abundance in pHNAH67 (copy number/μL)
	MT[a]	Standard Curve	Amplification Efficiency (%)	R^2	
floR	60	C=−.135×q+41.743	108.4	0.990	5.093 × 10^8
sul2	60	C=−3.584×Cq+42.318	90.1	0.995	1.162 × 10^8
aph(4)-Ia	60	C=−3.645×Cq+45.090	88.1	0.990	4.015 × 10^8
aac(3)-IV	60	C=−3.511×Cq+41.778	92.7	0.998	5.385 × 10^8
fosA3	60	C=−3.553×Cq+39.757	91.2	0.998	1.905 × 10^8
bla$_{CTX-M-65}$	60	C=−4.511×Cq+53.805	66.6	0.994	2.378 × 10^8
aac(6′)-Ib-cr	60	C=−3.476×Cq+42.394	93.9	0.994	3.958 × 10^4
bla$_{OXA-1}$	60	C=−3.709×Cq+45.542	86.1	0.991	2.826 × 10^4
catB3	60	C=−3.099×Cq+37.961	110.2	0.994	6.052 × 10^3
arr3	60	C=−3.148×Cq+39.218	107.8	0.991	3.943 × 10^3
sul1	60	C=−3.388×Cq+41.137	97.3	0.993	2.160 × 10^8

The qPCR procedure was as follows: initial 95 °C denaturation for 1 min, followed by 40 cycles consisting of denaturation 95 °C for 10 s, annealing for 5 s at melting temperature shown above with plate read, extension 72 °C for 15 s and a final melt curve step from 65 to 95 °C in increment of 0.5 °C for 5 s with plate read. The concentration of plasmid DNA was 49.1 ng/μL. [a]Melting temperature (°C).

Table S4. Results of amino acid sequence alignments of tail fiber, ribonucleotide reductase and holin between Enterobacteria phage T4 or RB3 and phage AH67C600_Q9[a.]

Amino acid sequence	Gene Position on Phage AH67C600_Q9 Genome (bp)	Identity (%)	
		T4	RB3
Tail fiber	70617–70859	97.50	94.67
	129359–129595	96.15	92.31
	145953–149822	95.50	92.55
	151683–154781	56.85	72.00
Ribonucleotide reductase	40154–40624	80.77	99.23
	135747–136925	86.84	85.71
	136979–139234	86.82	88.02
Holin	155373–156032	75.34	74.43

[a]Amino acid sequence alignments with identities <95%.

Table S5. Matching positions of contigs to ARGs for *E. coli* AH67C600.

Gene	Plasmid Position	Reads Number	Contigs-Length	BLAST Position
floR	84882–86095	32	contig_941_655bp	85314-85951
sul2	88599–89414	21	contig_914_575bp	88375-88949
aph(4)-Ia	94261–95286	47	contig_406_625bp	94262-94808
aac(3)-IVa	95507–96291	7	ND[α]	ND
fosA	98920–99336	21	contig_41_903bp	98119-99021
bla$_{CTX-M-65}$	102052–102927	15	ND	ND
aac(6′)Ib-cr	103968–104567	0	ND	ND
bla$_{OXA-47}$	104698–105528	4	ND	ND
catB3	105666–106298	0	ND	ND
ARR-3	106346–106835	0	ND	ND
sul1	107312–108238	12	ND	ND

[α] Not detected.

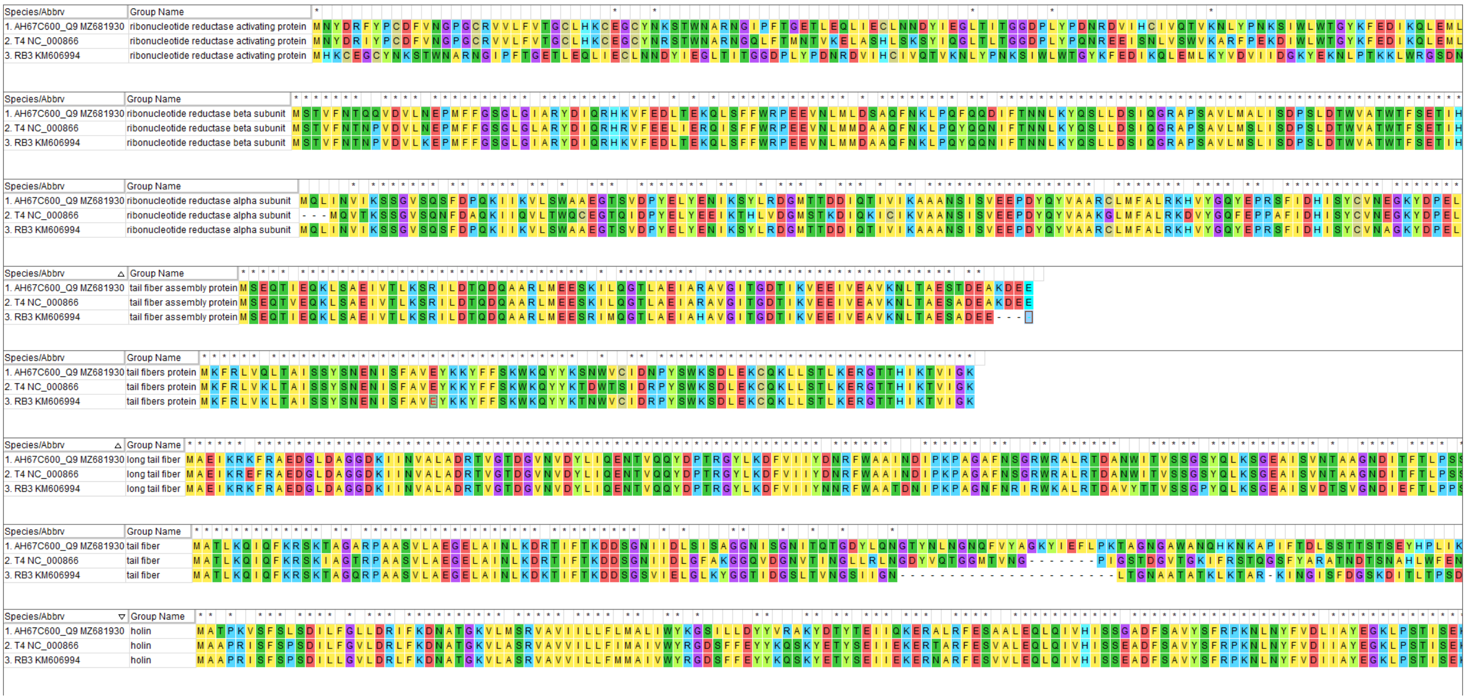

Figure S1. Amino acid sequence alignments of tail fiber, ribonucleotide reductase and holin between Enterobacteria phage T4 or RB3 and phage AH67C600_Q9. This figure only shows the results of the amino acid sequence alignment identity are less than 95% (Table S4). At the same position in different sequences, amino acid differences were indicated by different colors, while the same color indicated the same or similar amino acids. The asterisk above indicated that the amino acid at this position is conserved.
